# A Randomised, Double Blind Trial of N-Acetylcysteine for Hearing Protection during Stapes Surgery

**DOI:** 10.1371/journal.pone.0115657

**Published:** 2015-03-12

**Authors:** Dan Bagger-Sjöbäck, Karin Strömbäck, Pierre Hakizimana, Jan Plue, Christina Larsson, Malou Hultcrantz, Georgios Papatziamos, Henrik Smeds, Niklas Danckwardt-Lillieström, Sten Hellström, Ann Johansson, Bo Tideholm, Anders Fridberger

**Affiliations:** 1 Center for Hearing and Communication Research, Department of Clinical Science, Intervention, and Technology, Karolinska Institutet, SE-171 77 Stockholm, Sweden; 2 Department of Otolaryngology, Karolinska University Hospital, SE-171 76 Stockholm, Sweden; 3 Department of Otolaryngology, Academic Hospital, SE-751 85 Uppsala, Sweden; 4 Department of Physical Geography and Quaternary Geology, Stockholm University, SE-106 91 Stockholm, Sweden; 5 Department of Audiology and Neurotology, Karolinska University Hospital, SE-171 76 Stockholm, Sweden; 6 Department of Clinical and Experimental Medicine, Linköping University, SE-581 85 Linköping, Sweden; University of Regensburg, GERMANY

## Abstract

**Background:**

Otosclerosis is a disorder that impairs middle ear function, leading to conductive hearing loss. Surgical treatment results in large improvement of hearing at low sound frequencies, but high-frequency hearing often suffers. A likely reason for this is that inner ear sensory cells are damaged by surgical trauma and loud sounds generated during the operation. Animal studies have shown that antioxidants such as N-Acetylcysteine can protect the inner ear from noise, surgical trauma, and some ototoxic substances, but it is not known if this works in humans. This trial was performed to determine whether antioxidants improve surgical results at high frequencies.

**Methods:**

We performed a randomized, double-blind and placebo-controlled parallel group clinical trial at three Swedish university clinics. Using block-stratified randomization, 156 adult patients undergoing stapedotomy were assigned to intravenous N-Acetylcysteine (150 mg/kg body weight) or matching placebo (1:1 ratio), starting one hour before surgery. The primary outcome was the hearing threshold at 6 and 8 kHz; secondary outcomes included the severity of tinnitus and vertigo.

**Findings:**

One year after surgery, high-frequency hearing had improved 2.7 ± 3.8 dB in the placebo group (67 patients analysed) and 2.4 ± 3.7 dB in the treated group (72 patients; means ± 95% confidence interval, p = 0.54; linear mixed model). Surgery improved tinnitus, but there was no significant intergroup difference. Post-operative balance disturbance was common but improved during the first year, without significant difference between groups. Four patients receiving N-Acetylcysteine experienced mild side effects such as nausea and vomiting.

**Conclusions:**

N-Acetylcysteine has no effect on hearing thresholds, tinnitus, or balance disturbance after stapedotomy.

**Trial Registration:**

ClinicalTrials.gov NCT00525551

## Introduction

Otosclerosis is a common disorder [1,2] where the stapes gradually fuses with the surrounding bone, producing conductive hearing loss. Surgery is a well-established treatment option, where the diseased stapes is bypassed by a prosthesis. This improves low-frequency hearing by 25–30 dB, but high-frequency hearing often suffers [3–10]. For instance, in the study by Bauchet St. Martin et al [6], the average air conduction thresholds at 8 kHz were 8 dB worse six weeks after surgery. Improvement was seen in most patients over time, but an average decline of 4 dB remained at 9 months. Similarly, patients examined by Mair and Laukli [5] had 7 dB deterioration in hearing at 8 kHz one year after surgery; high frequency audiometry demonstrated a 15-dB average worsening of hearing thresholds at 10 kHz and even larger losses at 12 and 14 kHz. In Bauchet St Martin’s study [6], 49% of patients had worse hearing at 8 kHz 9 months after surgery. Strömbäck et al [3] used a more conservative measure, the average change in hearing across the three frequencies 4, 6, and 8 kHz, and found that 6.5% of patients had threshold deterioration larger than 10 dB after surgery. Finally, recent studies [11] that extended the frequency range of bone conduction measurements demonstrated that 8-kHz thresholds are worse in 54% of patients six weeks after surgery; 25% of patients had bone conduction threshold losses that were 10 dB or larger. These studies indicate that deterioration of high-frequency hearing occurs in a substantial fraction of patients undergoing stapes surgery, despite the great benefit observed at lower frequencies.

High-frequency loss after stapes surgery may be a result of surgical trauma, perilymph leakage, and loud sounds generated by drilling close to the delicate sensory cells in the inner ear [3,6,12]. Studies suggest that pharmacological intervention can target some of these factors. For instance, loud sound causes toxic free radicals to be generated in the hearing organ [13] and animal studies consequently demonstrate that hearing loss induced by noise can be decreased by antioxidants, such as N-Acetylcysteine [14–17]. N-Acetylcysteine has also shown some effectiveness against surgical inner ear trauma in animal models [18].

It is not known if the human ear can be protected by antioxidants, but N-Acetylcysteine is nonetheless incorporated in treatment programs for noise-exposed soldiers, and marketed as a nutritional supplement for protecting the ear against noise [19].

Hearing loss that follows from noise or other trauma may be temporary or permanent. The temporary component can last for up to four weeks and is thought to depend on mechanisms other than free radical generation [20]. Nevertheless, three small, short-term, randomized studies tested the effect of N-Acetylcysteine on temporary threshold shifts in humans [21–23]. Minor but significant effects were observed in one of these trials [21], but the effect of antioxidants on permanent threshold shifts have not been examined.

We performed a clinical trial to determine if N-Acetylcysteine can improve high-frequency hearing (6 and 8 kHz) after stapedotomy. Patients were followed for one year after surgery, using audiograms as well as questionnaires designed to probe the severity of tinnitus and balance disturbance and the general satisfaction with surgery.

## Methods

### Study design, oversight, and ethics statement

This randomized, placebo-controlled, and double-blind parallel-group trial was performed at three university clinics in Sweden. The first and last author designed the study with input from remaining authors. The protocol and patient consent forms were approved by the Regional Ethics board in Stockholm (2007/305-31/4; S1 CONSORT Checklist S1 and S1 Protocol) and by the Swedish Medical Products Agency (151:2007/17252); the study was registered with ClinicalTrials.gov (accession number NCT00525551) and EudraCT (2006-006243-31). Data were analysed by two statistical consultants (PH and JP) and by the last author. The report was written by the last author, the other authors providing comments and suggestions on several previous drafts as well as approval of the final version.

### Eligibility

Eligible patients had a clinical diagnosis of otosclerosis (defined as typical history and audiogram, air-bone gap of at least 20 dB at 0.5, 1, 2 and 3 kHz, and normal middle ear status without signs of infection or perforation of the eardrum), had opted for surgical treatment, were older than 18 years of age, and had signed the consent form. We excluded patients with asthma [24], hypersensitivity to the study drug, only one functioning ear, and those with previous middle ear surgeries. In premenopausal patients not using hormonal anticonception or intrauterine devices, serum chorionic gonadotropin was measured before administering the study drug; levels >5 IU / l led to exclusion.

### Study procedures

Hearing was measured in soundproof booths with patients wearing calibrated TDH 39 headphones. Pure tones at 0.25, 0.5, 1, 2, 3, 4, 6 and 8 kHz were presented to one ear at a time. Thresholds for bone-conducted sound (0.25, 0.5, 1, 2, 3 and 4 kHz) were measured by placing a calibrated vibrator on the mastoid process. Patients indicated stimulus perception by pressing a button; the threshold was identified using the modified Hughson-Westlake method as recommended by the International Standards Organization (ISO8253-1). Speech discrimination was tested using a set of 50 phonemically balanced words presented at the patient’s most comfortable level (commonly located 30 dB above the average hearing threshold at 0.5, 1, and 2 kHz).

Patients graded the severity of tinnitus during the past week using a rating scale where zero was defined as no tinnitus and 10 as a high degree of disturbing tinnitus. A similar strategy assessed subjective vertigo. Satisfaction with hearing quality was estimated using a scale where zero corresponded to very low and ten indicated very high satisfaction.

The measures listed above were performed before surgery, 6–8 weeks thereafter, and at one year. At the last two time points, patients also graded the overall outcome, zero corresponding to very poor and 10 indicating a very good result.

On the evening after surgery, physicians scored facial nerve function and gustation as normal or abnormal and nystagmus as present or absent. Patients also scored tinnitus and vertigo at this time point.

### Randomisation and masking

Subjects eligible for the study were divided in four strata based on age (over or under 45 years) and hearing threshold for bone-conducted sound at 4 kHz (better or worse than 40 dB hearing level). Stratified randomization with a block size of 6 was used to assign patients to intravenous N-Acetylcysteine (150 mg/kg body weight, mixed with physiological saline to a final volume of 300 ml) or matching placebo (300 ml of identical-appearing saline solution; the allocation ratio was 1:1). Infusions started one hour before surgery at a rate of 100 ml/h. The randomization lists were computer-generated by the pharmacy prior to the start of the study and kept at a central facility. Patients and all personnel involved in the study were unaware of treatment assignments.

### Surgery

Eight experienced otologic surgeons performed operations. Surgeons were free to choose the technique they deemed best. Hence, in 35 of 156 cases a laser was used for thinning the stapes footplate and for vaporizing the tendon and the posterior and anterior crus of the stapes, and a skeeter microdrill utilized for stapedotomy. A microdrill was used throughout surgery in remaining cases. Periauricular infiltration of lidocaine-epinephrine was used in all patients; 10% also received general anaesthesia. Sedatives and antiemetics were used liberally, including betamethasone in 17 patients in the actively treated group and 25 patients in the placebo group. A separate statistical analysis showed that betamethasone lacked effect on primary or secondary outcomes. A teflon / platinum Fisch prosthesis with 0.4 mm diameter was used in 98% of cases.

### Outcomes

In animal experiments, N-Acetylcysteine protects from loss of sensory cells but is less effective against the reversible hearing impairment that follows immediately after trauma [25]. Because the 6–8 week data may reflect temporary effects, the primary outcome was the hearing threshold at 6 and 8 kHz one year after surgery.

Secondary measures of efficacy included hearing thresholds at those frequencies 8 weeks after surgery, and the severity of tinnitus and vertigo at these two time points. Tinnitus was included as a secondary outcome measure because of its well-established relation to high-frequency hearing loss [26], and balance problems appear to be more common in patients where the inner ear is subject to greater surgical trauma.

To assess safety, we monitored facial nerve problems, gustation loss, and nystagmus, and the incidence of side effects from N-Acetylcysteine, such as exanthema, urticaria, bronchospasm, and anaphylactoid reactions [27].

### Statistical analysis

Estimations showed that 150 patients would result in 90% power to detect an intergroup difference in hearing thresholds of 10 dB at a significance level of 0.05. A protocol-specified interim analysis was conducted after recruitment of 75 patients; this analysis showed no significant intergroup difference. The trial therefore continued until the number of patients dictated by the power analysis had been reached.

Data were analysed using linear mixed models with Gaussian error distribution [28]. To correct for patient age, sex, weight and the fact that the postoperative hearing recovery depended on stimulus frequency, we included these terms as independent variables. Pearson correlation, ANOVAs and χ2-tests were used to test for collinearity among predictor variables. T-tests, the Wilcoxon rank sum test and the χ2-test were used to compare baseline patient characteristics.

Linear mixed models were used to analyse the effect of medical treatment on the change in hearing thresholds after surgery. To account for the autocorrelation arising from the longitudinal data collection performed during audiometry, a patient-specific intercept was used as the random effect, and predictor variables mentioned above treated as fixed effects. Likelihood ratio testing (χ2 test) was used to construct the optimal linear mixed model by starting from a full model and removing model terms in a stepwise manner. Finally, a likelihood ratio test compared the null model (including patient-specific intercept) to the final model, to evaluate the significance of adding that set of predictor variables. The intercept was significant (p < 0.001), confirming the appropriateness of the linear mixed model.

Tinnitus, dizziness and the perceived quality of hearing were analysed using ordered logistic regression. The dependent variable was the change in scores as compared to baseline. All analyses were performed in the R Environment for Statistical Computing (v2.12.1).

## Results

### Patients

**Fig 1:**
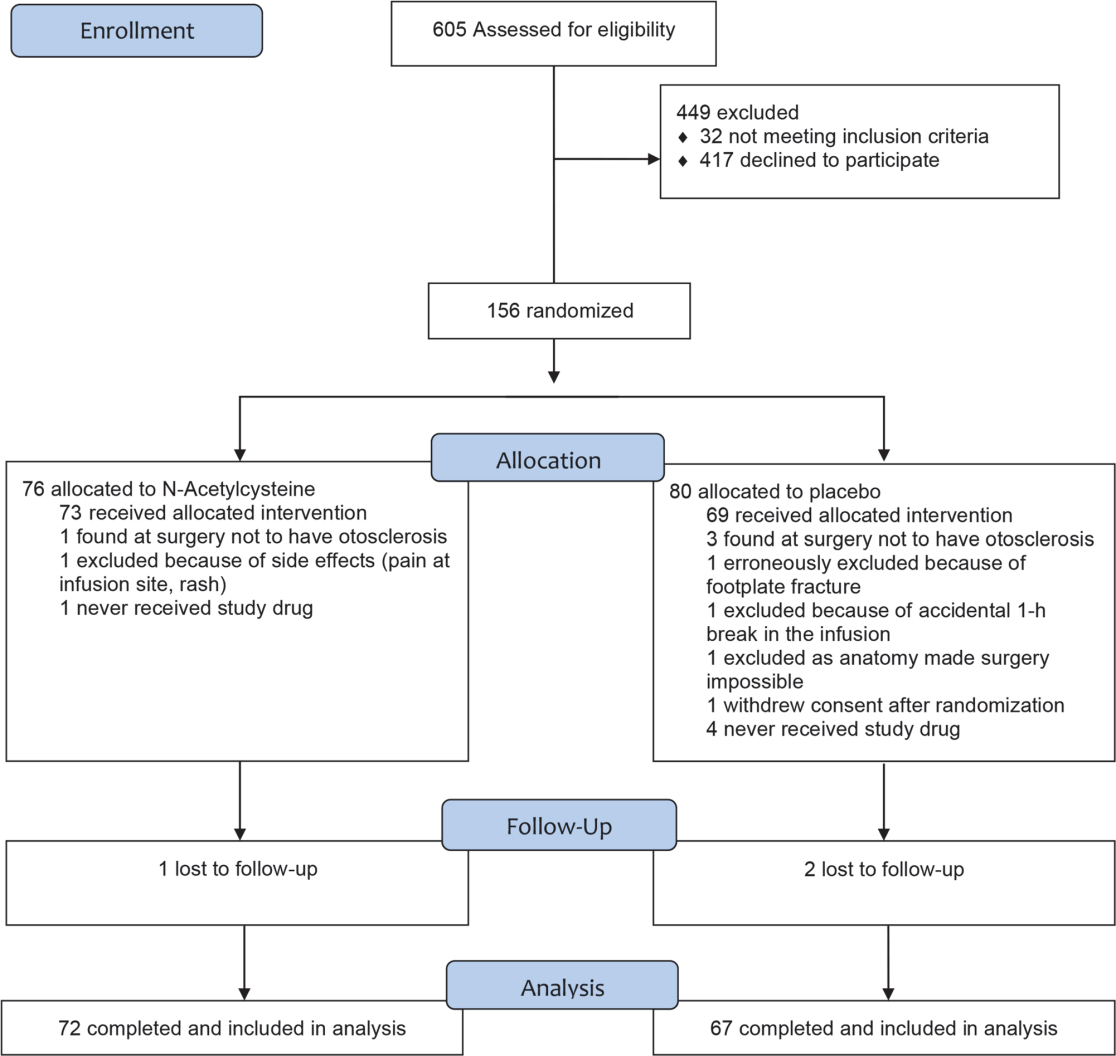
Consort flow diagram. A common reason for declining trial participation was that long travel distances made patients unwilling to return to the hospital for the 1-year follow-up visit. Otosclerosis is a clinical diagnosis, and 4 patients were found at surgery to have middle ear disorders where stapedotomy is not indicated. One patient in the placebo group was excluded in violation of the protocol because of a fracture of the stapes footplate. A total of 5 patients never received the study drug because their operations were postponed after study drugs had been delivered to the ward (but before drug infusion started).

Between November 2007 and April 2012, 605 patients with otosclerosis were assessed for eligibility (Fig. 1). From these patients, 26% (156 patients) were randomized to either N-Acetylcysteine or matching placebo. Data for the primary outcome was available for 139 subjects (89%); 72 patients in the treated group and 67 in the placebo group finalized the study and were included in statistical analyses. A common reason for dropouts was that patients lived far from the clinic and were unwilling to travel.

Patient characteristics are given in Table 1 Age, sex, and weight did not differ significantly between groups. Both groups rated their hearing as poor; some patients also suffered from tinnitus, while balance problems were uncommon before surgery. There were no significant intergroup differences in any of these parameters.

**Table 1:** Demographic characteristics, hearing, and surgical factors in study patients.

	NAC group (mean ± 95% CI, n = 72)	Placebo group (mean ± 95% CI, n = 67)	
**Demographic characteristics**			
Age—yr	49.1 ± 2.7^NS^	50.0 ± 2.8	t-test
Female sex—no (%)	48 (65) ^NS^	48 (68)	χ2-test
Weight—kg	72.9 ± 3.6 ^NS^	73.5 ± 3.2	t-test
**Hearing**			
Hearing threshold for bone-conducted sound at 4 kHz—dB HL	22.5 ± 3.6 ^NS^	21.3 ± 4.0	t-test
Air-Bone gap—dB	29.3 ± 2.0 ^NS^	29.1 ± 2.2	t-test
Speech comprehension—%^1^	94.8 ± 1.3 ^NS^	93.5 ± 2.4	t-test
Perceived hearing quality before surgery^2^	4 (0–9) ^NS^	5 (0–9)	Wilcoxon
Tinnitus grade before surgery^3^	3 (0–10) ^NS^	3 (0–10)	Wilcoxon
Dizziness grade before surgery^3^	0 (0–4) ^NS^	0 (0–8)	Wilcoxon
**Surgical characteristics**			
Complicated surgery^4^—no (%)	16 (22) ^NS^	10 (14)	χ2-test
Fractured footplate—no (%)	1 (1.4) ^NS^	1 (1.4)	χ2-test
Chorda tympani lesion—no (%)	2 (2.7) ^NS^	1 (1.4)	χ2-test
Problematic facial nerve anatomy—no (%)	4 (1.4) ^NS^	2 (2.8)	χ2-test
Bleeding—no (%)	1 (1.4) ^NS^	2 (2.8)	χ2-test
Duration of surgery—min	51.5 ± 5.6 ^NS^	50.3 ± 3.2	t-test
Prosthesis length—mm	4.68 ± 0.1 ^NS^	4.58 ± 0.1	t-test
Change in gustation^5^—no (%)	11 (15) ^NS^	14 (20)	χ2-test
Facial nerve dysfunction^5^—no (%)	0 (0) ^NS^	2 (3)	χ2-test
Nystagmus^5^—no (%)	2 (3) ^NS^	0 (0)	χ2-test
Perioperative tinnitus grade^3^	1 (0–10) ^NS^	1 (0–10)	Wilcoxon
Perioperative dizziness grade^3^	3 (0–10)*	1 (0–10)	Wilcoxon

N.S. denotes not significant.

* denotes P<0.05.

1. Measured with a standard 50-item phonemically balanced word list

2 Measured with a 10-grade scale where 10 represent the best possible hearing quality. Numbers are medians and ranges.

3. Measured with a 10-grade scale where 10 represents very disturbing tinnitus or dizziness. Numbers are medians and ranges.

4 As reported by the surgeon performing the operation.

5 Reported by physician after examining the patient on the evening after the operation.

The largest benefit from drug treatment is expected when surgery is complicated, since this may be associated with greater trauma. Surgeons therefore rated each operation as complicated or uncomplicated, and provided details in cases deemed difficult. There were no significant intergroup differences in the number of complicated surgeries, duration of the operation, or length of the implanted prosthesis. The most common perioperative problem was transient gustation change, which affected 15–20% of patients, and dizziness. Dizziness increased immediately after the operation with the N-Acetylcysteine group reporting more severe balance disturbance (p = 0.045, Wilcoxon rank sum test).

**Fig 2:**
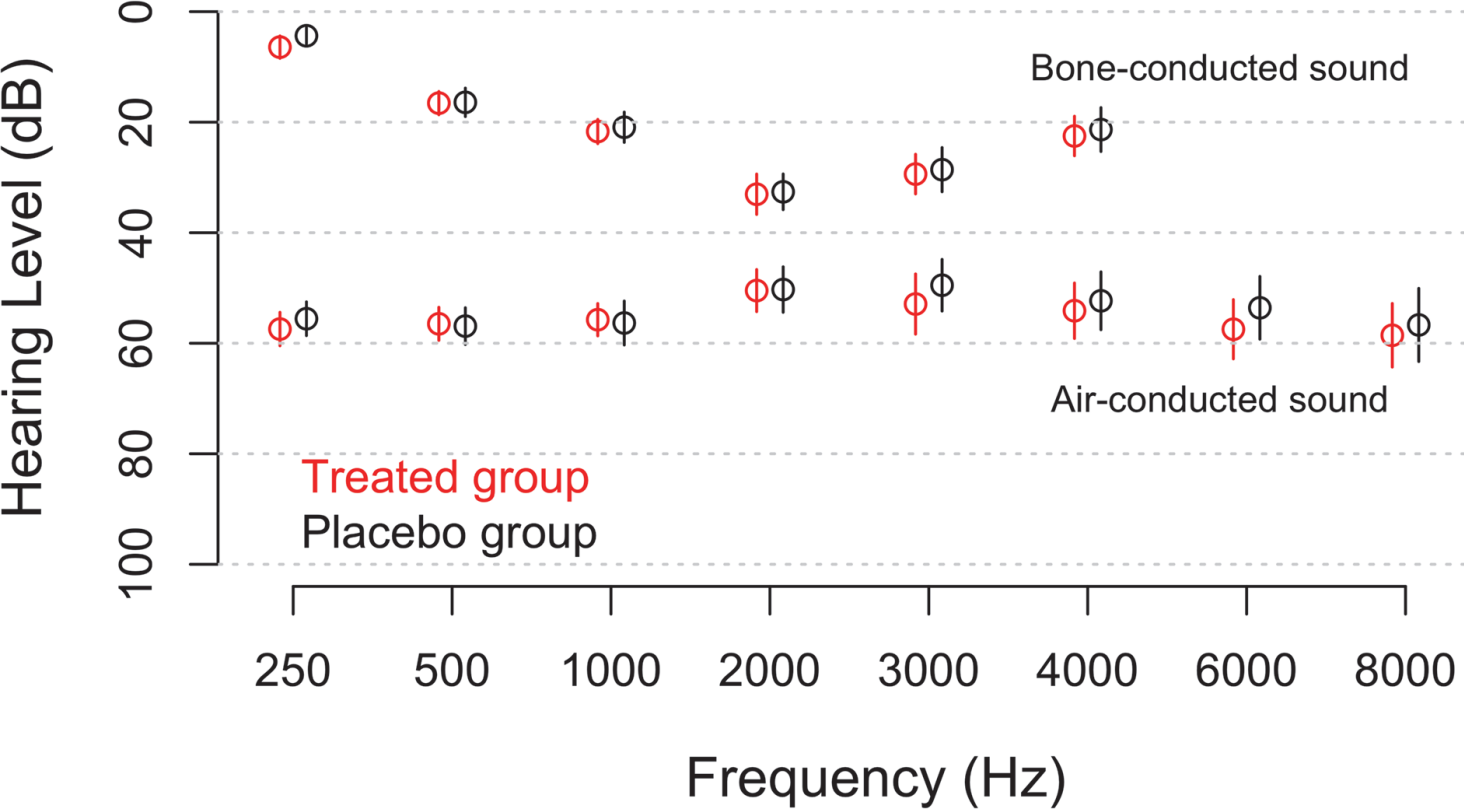
Hearing thresholds of the placebo group and the N-Acetyl cysteine group (“Treated group”) at baseline. Thresholds for air-conducted sound (lower set of points) are measured by standard headphones covering the ears; thresholds for bone-conducted sound (top data set) are determined by placing a vibrator on the mastoid process. Bone-conducted sound was not measured at frequencies higher than 4000 Hz as this is not part of normal clinical routine. Rings represent the mean values; vertical lines are 95% confidence intervals for the mean. Hearing was measured at the same set of frequencies in all patients, but a horizontal offset was added when plotting to improve the clarity of presentation.

Before surgery, thresholds for air-conducted sound (Fig. 2, lower set of points) reveal a flat loss around 60 dB HL, whereas bone-conduction thresholds (top set of points in Fig. 2) have the notch pattern common in otosclerotic ears; the mean preoperative air-bone gap was 29 dB. Hearing did not differ significantly between the groups.

Overall, the two groups were well balanced with regard to patient characteristics, parameters reflecting surgical difficulties, and hearing.

### Outcomes

**Fig 3:**
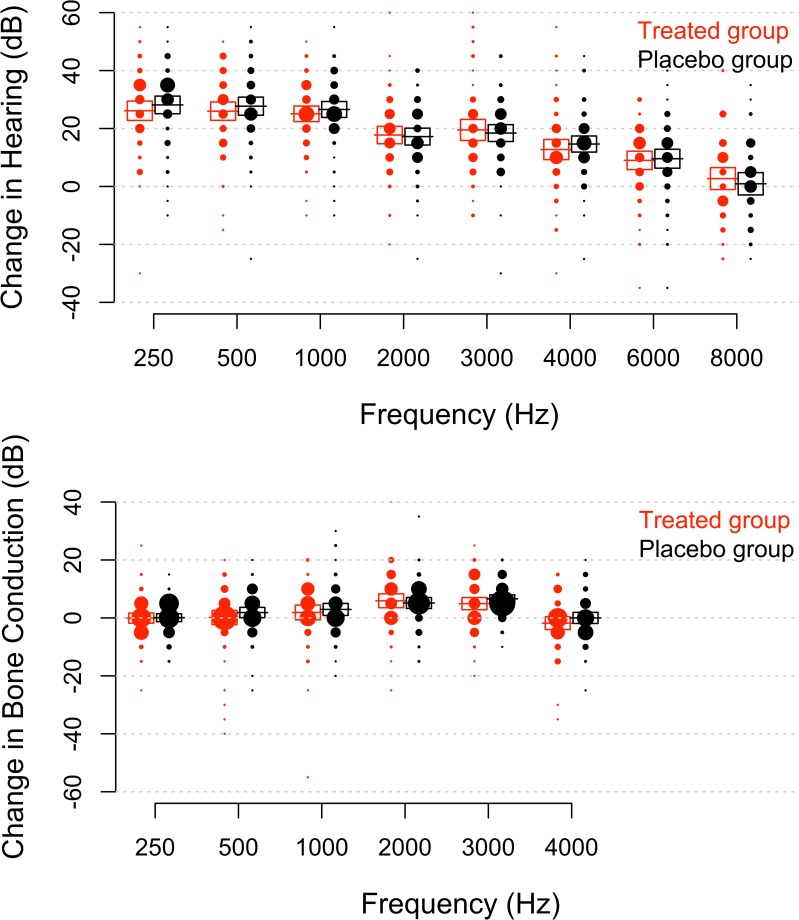
(Upper graph) Change in hearing thresholds 6 weeks after surgery (as compared to measurements before the operation). Hearing was measured at the same set of frequencies as in Fig. 2. The size of each dot corresponds to the number of patients, obtained from the histogram for each frequency (5 dB bins) and boxes enclose the 95% confidence interval. Horizontal lines show the mean value at each frequency. One patient with complete sensorineural hearing loss after surgery is omitted from the plot (but included in all statistical analyses). **(Lower graph)**. Change in bone-conduction thresholds at 6 weeks. The scaling of the dots is the same in both panels.

Stapedotomy improved low-frequency hearing in both groups. At 250 Hz, patients receiving N-Acetylcysteine gained 26 ± 3.3 dB and those given placebo 28 ± 3 dB at 6 weeks (means ± 95% confidence intervals; Fig. 3; the standard deviation was 14 and 13 dB, respectively). The benefit from surgery decreased at higher frequencies (p<0.001; slope = −0.0032 dB / Hz; linear mixed model) and at 8 kHz, the average change was 2.7 ± 3.8 dB in the treated group and 0.9 ± 3.8 dB in the placebo group (standard deviation 16 dB in both groups). At this frequency, 34% of patients in the placebo group and 43% of those in the treated group lost hearing after surgery. The loss was 15 dB or larger in 19% of patients. At 6 kHz, 17% of patients in the placebo group and 21% of those in the N-Acetylcysteine group lost hearing; the loss was 15 dB or larger in 9%. The linear mixed model indicated a non-significant negative effect from N-Acetylcysteine (p = 0.69).

**Fig 4:**
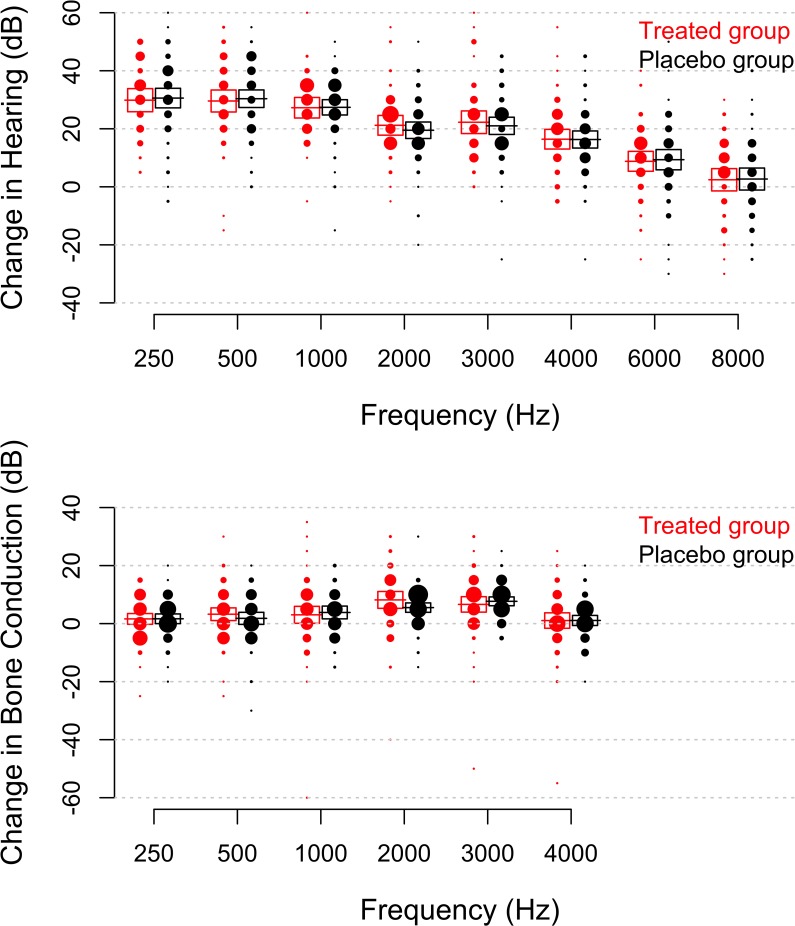
(Upper graph). Change in hearing thresholds one year after surgery (as compared to measurements obtained before the operation). The size of each dot corresponds to the number of patients (obtained from the histogram for each frequency; 5 dB bins). Boxes enclose the 95% confidence interval and the horizontal lines give the mean at each frequency. One patient with complete sensorineural hearing loss after surgery is omitted from the plot. (**Lower graph**). Change in bone-conduction thresholds at 1 year. Dot scaling is identical in both graphs.

The improvement at one year (Fig. 4) correlated to the improvement observed at 6 weeks, which depended on frequency. To avoid predictor variable collinearity, we analysed the difference in hearing thresholds between these two time points. A small additional improvement, most pronounced at low frequencies, was evident during the first year (model slope −0.0003 dB / Hz; p<0.001; linear mixed model), but N-Acetylcysteine had no significant effect (p = 0.68; 38% of patients in the placebo group and 36% of patients in the treated group lost hearing at 8 kHz; the corresponding values at 6 kHz were 20 and 21%).

In exploratory analyses not specified in the protocol, we computed the number of patients who lost hearing at most frequencies. In each group, 4 patients had average change in hearing less than 0 dB across all frequencies (P = 1, χ2-test). One patient in the N-Acetylcysteine group had complete sensorineural hearing loss; there were no deaf ears in the placebo group.

### Other outcomes

Stapedotomy decreased tinnitus severity. At both 6 weeks and 1 year, tinnitus scores were about one unit better (on a 10-grade scale; Table 2), but there was no significant difference between the groups. The improvement was evident soon after surgery, with a small further gain over the first year.

**Table 2:** Changes in scores for tinnitus, dizziness, and other variables.

	NAC group (n = 72)	Placebo group (n = 67)	
Change in tinnitus grade from baseline to 6 weeks^6^	0.99 ± 1.2^NS^	0.93 ± 1.5	OLR
Change in tinnitus grade from baseline to 1 year^6^	1.07 ± 1.1^NS^	1.24 ± 1.5	OLR
Change in dizziness grade from baseline to 6 weeks^6^	−0.07 ± 0.7*	−0.42 ± 0.8	OLR
Change in dizziness grade from baseline to 1 year^6^	1.72 ± 0.9^NS^	1.88 ± 0.8	OLR
Change in hearing quality from baseline to 6 weeks^6^	2.3 ± 1.2*	3.1 ± 1.4	OLR
Change in hearing quality from baseline to 1 year^6^	2.67 ± 1.2^NS^	3.31 ± 1.3	OLR
Satisfaction with surgery at 6 weeks^7^	7.0 ± 1.3^NS^	7.7 ± 1.2	OLR
Satisfaction with surgery at 1 year^7^	7.9 ± 1.3^NS^	8.2 ± 1.1	OLR
Air-bone gap at 6 weeks—dB^8^	10.3 ± 1.4^NS^	10.8 ± 1.6	t-test
Air-bone gap at 1 year—dB^8^	10.8 ± 1.7^NS^	11.3 ± 1.7	t-test

N.S. denotes not significant; OLR, ordered logistic regression.

* = p < 0.05

6. Positive changes denote improvement on the 10-grade scale used in Table 1.

7. Patients graded the outcome on a 10-grade scale where 10 corresponded to a very good outcome and 0 to a very poor one.

8. The air-bone gap is the average difference between air conduction thresholds and bone conduction ones at the frequencies 0.5, 1, 2, and 3 kHz. Data are means ± 95% CI.

Many patients experienced vertigo on the day of surgery (see Table 1), an effect that remains noticeable at 6 weeks (Table 2), where scores indicated a small residual vertigo sensation that was more pronounced in the placebo group. This intergroup difference was borderline significant (p = 0.048, ordered logistic regression) but vertigo improved over the course of the first year and on average, patients reported less dizziness at 1 year than they did before surgery. This improvement was similar in both groups.

When grading their hearing quality, patients reported a substantial improvement after surgery. The improvement was larger in the placebo group at both time points; this difference was borderline significant at 6 weeks (p = 0.047), but not at 1 year (p = 0.051). A similar trend was evident for the general satisfaction with surgery, where the average was an 8 out of 10 grading at one year, without significant differences between groups.

Air-bone gaps were stable over the course of the first year. At one year, 57% of patients in the placebo group and 62% of patients in the treated group had air-bone gap less than or equal to 10 dB. This difference was not significant.

### Side effects

Four patients, all in the N-Acetylcysteine group, experienced side effects (vomiting, flush, thrombophlebitis, and pain at the infusion site). Pain at the infusion site led one patient to withdraw consent; the pain subsided promptly upon termination of the infusion. The remaining three patients completed the study.

## Discussion

Although stapes surgery improves low-frequency hearing by 25–30 dB on average, it is well established that this operation also can lead to sensorineural hearing loss. The incidence of profound hearing loss after stapes surgery appears to be 0.2–1% [9], but the present study was not powered to detect a difference in the incidence of such rare events. However, it is also clear that surgical results are worse at stimulus frequencies >4 kHz than they are at low frequencies [4–7]; a recent study showed that 54% of patients had decline in bone-conduction thresholds at 8 kHz six weeks after surgery [8]. The relatively poor high-frequency results may be an effect of surgical trauma and loud sounds generated during perforation of the stapes footplate. Some patients describe the sound of the microdrill as the “worst I ever heard”, and attempts at measuring the sound pressure generated by drilling has shown values in the range between 117 and 131 dB SPL [9], which is loud enough to cause significant hearing loss. Barotrauma, accidental suctioning of perilymph, and leakage of perilymph during prosthesis insertion may also contribute [6,8]. These trauma-related components are a potential target for pharmacological interventions.

Animal studies showed that many pathways can be manipulated in order to protect inner ear sensory cells [29]. Antioxidants have shown significant promise, with a protective effect on the order of 10–30 dB after standardized noise trauma. N-Acetylcysteine has also shown some effectiveness against surgical trauma in animal models [15]. We therefore expected antioxidants to improve surgical results by modifying the effects of surgical trauma and any loud sound generated during footplate perforation. However, high-dose N-Acetylcysteine did not improve hearing thresholds or tinnitus. The perceived quality of hearing was worse in the N-Acetylcysteine group at 6 weeks, but this difference was only marginally significant and not present at 1 year. Vertigo was worse in the placebo group at 6 weeks, but again the difference was only marginally significant and not sustained. Given their inconsistency and low magnitude, it is likely that these differences were the result of chance.

Previous studies suggest that patients with normal or near normal high-frequency hearing thresholds have the highest risk of developing post-surgical hearing loss [3,6]. These patients may have normally functioning sensory cells at the base of the cochlea and a small loss of such cells may be sufficient to measurably decrease hearing. Hence, a protective effect from antioxidants could be present in this subgroup of patients, but the group is not large, making recruitment for such a trial a challenge.

Short follow-up times were used in all previous human trials of antioxidants for hearing protection [19–21]. This is problematic, because temporary threshold shifts are thought to depend on mechanisms where antioxidants are not expected to be optimally effective [18]. A long follow-uptime is therefore important, although we note that there were only minor changes in hearing between weeks 6 and 52 in the present study. Hence, future trials in this area could probably be restricted to a 6-week time window without significant loss of power.

Previous trials also used relatively low oral doses of antioxidants. In the case of N-Acetylcysteine, this is a problem because only 6–10% of an oral dose reaches the systemic circulation [30], and the half-life is approximately 2 h. Intravenous treatment was therefore used in the current study. Although it is difficult to extrapolate doses from animals to humans, the dose used here is within the range found effective in animals [10], and the average dose is around 9 times that used in human trials published to date [20,21].

Pharmacological inner ear protection is difficult to study in humans, since people cannot be exposed to loud sounds to determine if their ears will be protected. A potential solution is to study military personnel or other groups with frequent noise exposure, but hearing loss will often develop slowly even in these groups, which makes studies time-consuming, expensive, and prone to compliance problems. Stapes surgery may be a setting where the effects of protective drugs can be studied, since the operation leads to a temporally well-defined trauma on the high-frequency parts of the inner ear.

## Supporting Information

S1 CONSORT Checklist Consort 2010 checklist. (DOC)

S1 Protocol English translation of study protocol as approved by the Regional Ethics Board in Stockholm. (PDF)
