# Supplementary material for: A Randomised, Double Blind Trial of N-Acetylcysteine for Hearing Protection during Stapes Surgery
Source: PLoS One. 2015 Mar 12;10(3):e0115657. doi: 10.1371/journal.pone.0115657 (PMC4357436; doi:10.1371/journal.pone.0115657)
Supplement: S1 Protocol — (PDF) [file pone.0115657.s002.pdf]

Antioxidants during stapedotomy
English translation of study protocol KS-OAS1
Version 1.3 (8 May 2007)

Contents

Background and aims 4
Name and description of study drug 4
Previous studies 4
Potential risks of human use 4
Potential benefits 5
Dose, administration, and duration of treatment 5
Study population 6
Patient information 6
Efficacy endpoint 6
Study design 6
Randomisation and blinding 7
Inclusion criteria 7
Exclusion Criteria 7
Power 7
Time plan 8
Monitoring 8
Side effects 8
Data collection and statistical analysis 8
Access to data 9
Other treatments 9
Data 9
Insurance 9
Information to other personnel 9
Publication 10
References 10
Summary of substantial amendments to the study protocol 11

Antioxidants during stapedotomy
Study protocol KS-OAS1
Version 1.3 (May 8, 2007)

Principal Investigator:
Dan Bagger-Sjöbäck, M.D., Ph.D.
Professor of Otorhinolaryngology
Karolinska University Hospital Solna
171 76 Stockholm
e-mail: dan.bagger-sjoback@karolinska.se
Telephone: 08-51776032

Co-investigators:
Anders Fridberger. M.D.,PhD.
Associate Professor
Center for Hearing and Communication Research
Karolinska Institutet
M1 Karolinska University Hospital
171 76 Stockholm
e-mail: anders.fridberger@ki.se
08-51773274

Sten Hellström. M.D.,Ph.D.
Professor
Chairman, Dept. of Audiology and Neurotology
Karolinska University Hospital
171 76 Stockholm
e-mail:sten.hellstrom@karolinska.se
Telephone: 08-51776032

I hereby certify that this study will be conducted according to this protocol, in accordance with good clinical practice and applicable regulations

[Original protocol signed by Dan Bagger-Sjöbäck]

Dan Bagger-Sjöbäck
Principal Investigator

Background and aims

Otosclerosis is a disease of the ear that causes gradually increasing hearing loss. The stapes is gradually fixed inside the oval window, which leads to hearing impairment. The conventional treatment is surgical (stapedotomy): The stapes is partially replaced by drilling on its footplate and connecting a prosthesis. This results in great improvement of low-frequency hearing, but at frequencies above 4000 Hz, there is less improvement and in some cases even decline of hearing after surgery (see e.g. Meyer, 1999). This is considered a consequence of trauma to the high-frequency parts of the inner ear during drilling on the stapes footplate. During drilling, patients report loud sounds, often described as ”the worst sound I ever heard”.

In animals, antioxidants such as N-Acetylcysteine can protect the inner ear against noise-induced hearing loss (see below). We will examine whether antioxidants lead to improved high-frequency hearing after stapedotomy. Our hypothesis is that antioxidants protect the inner ear against the trauma caused by the operation.

Noise-induced hearing loss is a very common condition, which makes it important to determine whether pharmacological inner ear protection is feasible. The study is therefore important to many patients, not only those affected by otosclerosis.

Name and description of study drug

This study uses N-Acetylcysteine, a drug that has been registered for a very long time in Sweden. Approved indications for use include chronic bronchitis, cystic fibrosis, keratoconjunctivitis sicca, and paracetamole overdose. The drug also has antioxidant properties, which explains its planned use in the current study.

Previous studies

In animals, N-Acetylcysteine (and other antioxidants) can protect the inner ear from noise (see e.g. Ohinata et al, 2003; Duan et al 2004; Kopke et al 2005; Yamashita et al 2005). Animals given N-acetylcysteine prior to noise trauma had hearing thresholds 10 – 30 dB better than controls 3 – 4 weeks after sound exposure. This protective effect is seen after both continuous noise and intense impulse-like sounds.

Because humans cannot be intentionally exposed to loud sounds, it has not been possible to examine whether this protective effect applies in man. In a small American study (Kramer et al 2006), nicht-club visitors were given low-dose N-Acetylcysteine or matching placebo prior to entering the club. Hearing thresholds were measured before and after the visit. There were no differences in hearing thresholds, but the study has been criticized because the dose was very low, the noise exposure uncontrolled, and the number of subjects small.

Potential risks of human use

N-Acetylcysteine has been on the market for more than 30 years. During the years 1976 – 2005, 113 reports of possible side-effects concerning the intravenous formulation were submitted to the Swedish Medical Products Agency. A cause-effect relation was considered likely in 91 cases. The table below shows the most commonly reported side effects during this time period:

Side effect	Number of case reports
Exanthema	20
Urticaria	13
Angioedema	10
Itching	7
Stomatitis	6
Anaphylactic reaction	5
Diarrhea	5
Flush	5
Bronchospasm	2

During the time period, one death was regarded as connected to treatment with N-Acetylcysteine.

The side-effect spectrum of N-Acetylcysteine must be considered mild. A Danish study (Schmidt & Dalhoff 2001) examined 529 patients given intravenous N-Acetylcysteine as an antidote against paracetamole ovedose. Side effects were observed in 8.5% of patients, with a pattern similar to the table above. Skin effects were easily treated with antihistamines and steroids. More severe side effects such as bronchospasm and anaphylactic reactions occurred in 18 cases (3.5%), but these cases were easily treated and did not require intensive care. All side effects are more common in patients with asthma (relative risk 2.8).

Infusion of N-acetylcysteine with the protocol used for treating paracetamole overdose results in transient changes in plasma levels of certain coagulation factors. The levels of factor II, VII, and X decrease, but there is no effect on the APTT (Knudsen et al 2005).

It should be stressed that paracetamole overdose is a common clinical problem. The drug is therefore given intravenously to a large number of patients every year (approximately 1000 – 2000 / year in Sweden).

Potential benefits
N-Acetylcysteine will be used in the current study because of its potential hearing-protective effect, which is well established in animals. We hope that the same protective effect will benefit study patients and lead to considerably improved surgical results (see below). In the event that such an effect can be proven, it opens the possibility of treating acute noise-induced hearing loss. Noise-induced hearing loss affects about 10% of the population and frequently leads to communication problems as well as tinnitus and hyperacusis.

Dose, administration, and duration of treatment
N-Acetylcysteine is subject to extensive first-pass metabolism after oral dosing, resulting in bioavailability of only 6 – 10%. For this reason, intravenous treatment is used in the current study. Pharmacokinetic data shows an average half-life of around 2 hours (Borgström et al 1986). Animal studies show that the hearing-protective effect is most pronounced if the substance is given before trauma. We therefore plan to start the infusion one hour prior to surgery, to allow the drug to penetrate to inner ear sensory cells. Because free radical production is thought to continue for some time immediately after trauma, the infusion will be stopped one hour after the end of surgery. The

operation normally takes about 45 minutes. The dose is 150 mg / kg bode weight. This dose is dissolved in 0.9% NaCl to a final volume of 300 ml, resulting in a 100 ml/h infusion rate. Side effects from N-Acetylcysteine appear to be related to the infusion speed (Schmidt & Dalhoff 2001). The rate of infusion used here is considerably lower than the one used in treating paracetamole intoxication, where the same dose is given over 15 minutes. Patients will be given conventional pre-medication consisting of sedatives and antiemetics.

As placebo substance, physiological saline solution is used, given intravenously and at an identical volume and infusion rate. Assuming correct administration, this substance completely lacks side effects in the current population.

Study population
Patients at the Karolinska University Hospital undergoing stapedotomy because of otosclerosis.

Patient information
All patients scheduled for stapedotomy will receive written information about the study prior to their appointment with the surgeon. Additional information is given when meeting the physician. Informed consent is documented by patients and physicians signing the informed consent form, and by ticking the corresponding box in the CRF.

Efficacy endpoint
The primary efficacy endpoint is the hearing threshold, measured with pure tone as well as speech audiometry, one year after surgery. Secondary measures of efficacy include hearing thresholds 6 – 8 weeks after surgery and the severity of tinnitus and vertigo after surgery.

A small number of patients (<1%) experience complete deafness on the operated ear after surgery. While N-Acetylcysteine could conceivably reduce the occurrence of this complication, its low incidence means that we do not expect being able to prove an effect in the current study.

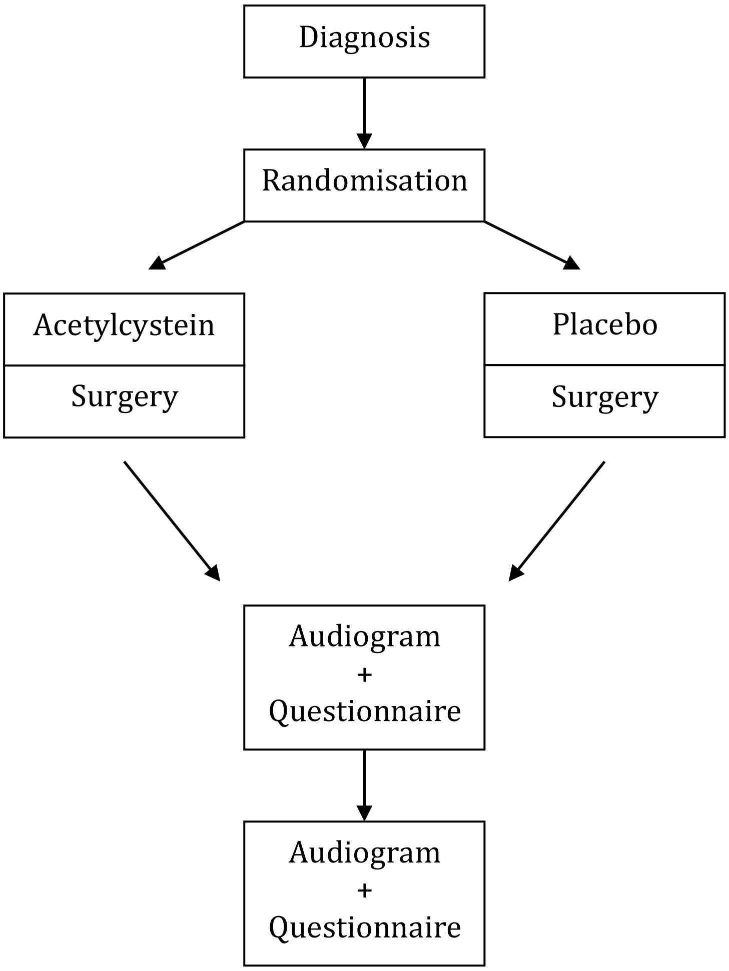

Study design
This is a randomized, placebo-controlled and double-blind study. After pure-tone audiometry and clinical diagnosis, patients are randomized to either active treatment or placebo. Before leaving

the hospital, study patients answer a questionnaire designed to determine the severity of tinnitus and vertigo. Pure-tone audiometry is repeated 6 – 8 weeks afters surgery. At this point in time, patients also grade their tinnitus and vertigo on a 10-grade visual analog scale. Patients are also asked to rate their satisfaction with the result of surgery. These measures are repeated one year after surgery.

Randomisation and blinding

Randomisation is handled by the Karolinska Pharmacy. To ensure that patients in the two arms of the study are similar with regard to age and pre-existing sensorineural hearing loss they are divided in four strata, based on age and hearing threshold for bone-conducted sound at 4 kHz, prior to randomization. Within each strata, block-wise randomization with 3 active / 3 placebo is used. Acetylcysteine and physiological saline solution have identical appearance. Exactly the same volume of each substance is given. Both substances are prepared, packaged, and labeled by the Karolinska Pharmacy.

Inclusion criteria

Patients must be older than 18 years of age and have otosclerosis diagnosed according to conventional clinical criteria (typical history and audiogram, air-bone gap of at least 20 dB and normal middle ear status without signs of infection or perforation). To be included in the study, patients should be candidates for surgery according to current criteria. Patients not fulfilling the above criteria are in such an early stage of their disease that surgery would not be an option, or they have other middle ear pathologies that make surgery impossible. Study patients are therefore expected to be highly representative for patients with otosclerosis scheduled for surgery.

Exclusion Criteria

Hypersensitivity to Acetylcysteine
Asthma
Previous bronchospasm after Acetylcysteine
Only one functioning ear
Reoperation
Pregnancy
Breast-feeding

Power

The smallest improvement of hearing thresholds considered clinically relevant is 10 dB. To have 90% power of capturing such an effect at 5% significance level, 55 patients per group are necessary (see figure 2, which was constructed based on the assumption of a 5 dB standard deviation in repeated audiogram measurements on the same patient). Animal studies show that Acetylcysteine gives 10 – 30 db improvement of hearing thresholds after impulse noise (Duan et al 2004; Kopke et al 2005).

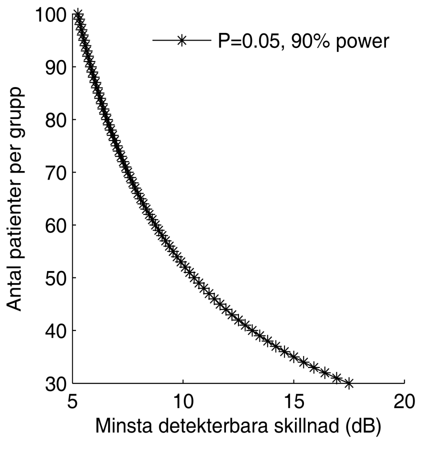

Figure 2. Relation between the number of patients per group and the smallest detectable difference in thresholds (90% power)

Data from the first 30 patients recruited will be used for a refined power calculation to determine whether a sample size of 110 patients is sufficient to

give the study the power to detect a smallest difference in hearing thresholds of 10 dB. No hypothesis testing will be performed at this stage, and later hypothesis testing can therefore be carried out without correction for this initial analysis.

Time plan
About 60 patients are operated every year at the Karolinska University hospital. Patient recruitment could therefore be completed in about 2 years, provided that the refined power analysis mentioned above shows the same result as the initial calculations.

All patients are followed for one year. Data collection should therefore be completed about 3 years after the start of the study. Statistical analysis and writing is projected to require about one year. This gives a total time span of about 4 years from the start of the study.

Monitoring
The study will be monitored by a specially trained nurse, Susanne Lagerqvist-Åhnblad. To capture problems at an early stage special attention will be given to the start of the study. When 3 – 5 patients have been recruited a first monitoring visit will be carried out to document adherence to the protocol, that informed consent has been properly documented, that the CRF is correctly filled out, and that the audiogram data in the CRF agrees with patient records at the Hearing and Balance clinic. Similar controls will be carried out every three months during the study.

Side effects
Acetylcysteine has no known late side effects. All side effects are therefore expected to occur during or immediately after the infusion. All patients are questioned by their physicians about side effects, and side effects are recorded on a special page of the CRF. All side effects requiring treatment will be promptly reported to the physician responsible for the study, whose pager- and mobile phone number is given on the CRF.

Data collection and statistical analysis
Hearing thresholds are measured according to the norms of the Hearing and Balance clinic. To decrease variability, all audiograms will be collected by one audiologist, Ann Johansson. Hearing thresholds for air-conducted sound are measured at the stimulus frequencies 250, 500, 1000, 2000, 3000, 4000, 6000, 8000, 10000, 12000, 16000 Hz. For each frequency, the change in hearing thresholds is computed by subtracting the value recorded postoperatively from the preoperative value. Acetylcystein is expected to affect hearing only at stimulus frequencies >5 kHz. Multiple linear regression, where the dependent variable is the average of the hearing thresholds above 5 kHz, will be used. Independent variables include patient age, preoperative hearing thresholds, and the treatment given (acetylcystein / placebo). Data from the first 30 patients will be used for a refined power calculation, but also to test this statistical model. The model will be adjusted according to the results of this calculation. No hypothesis testing will be performed at this stage.

Questionnaire data will be analysed with non-parametric tests.

An interim analysis is planned after recruiting 60 patients. The study will be terminated if this analysis shows $P<0.001$. If this is not the case, the study will continue until the

number of patients dictated by the power analysis have been recruited. The criterion for a significant effect is set to 0.049 in the second analysis. The overall result will be significant at approximately the 5%-level (0.999 x 0.951 ~ 0.95).

Patients without follow up at one year will be included in the model according to a "mixed model" where both follow-up time points will be handled together.

Access to data
This study was initiated by the people listed on the front page. Acetylcysteine has been on the market for many years and is a very cheap drug. There is therefore no commercial sponsor. Study personnel have unrestricted access to all study data.

Other treatments
Study patients are given the same treatment as all other patients operated for otosclerosis. The operation will be carried out in exactly the same way, using the same kind of prosthesis. These surgeries are usually performed in local anesthesia with the patient sedated. The choice of drugs is at the discretion of the treating physician and anesthesiologist in the usual dialog with the patient. The study places no restrictions on this. The infusion is given by trained personnel and compliance is therefore expected to be high.

Side effects of Acetylcysteine are treated by first stopping the infusion, second by the antihistamine clemastine (Tavegyl ®, slow intravenous injection of 2 mg during 2 - 3 minutes). If further treatment is necessary 8 mg of betamethasone will be given intravenously (Betapred ®). Anaphylactic reactions or bronchospasm are treated in the customary way (epinephrine and / or bronchodilators).

The hearing of the study patients will be measured also at frequencies higher than those used in normal clinical audiometry. This requires about 45 minutes of extra time per patient, but carries no risk and no discomfort to the patient.

Data
All data will be kept at the Ear-Nose-Throat clinic at the Karolinska University Hopsital in Solna, where a special storage area for data from previous clinical studies is available. Data will be saved for a minimum of 10 years after study completion.

Insurance
All patients are covered by the normal patient insurance, as well as the Swedish drug insurance.

Information to other personnel
All stapedotomies at the clinic are carried out by 3 surgeons, all of whom are informed about the study. Other physicians at the clinic are informed at the yearly research day of the clinic in May 2007. Anesthesiologists and ward personnel are informed before the start of the study.

Publication

The results of the study will be published in international scientific journals and possibly also in other media. These publications will all be written in such a way that individual patients cannot be identified.

References

Borgström L, Kagedal B, Paulsen O. (1986) Pharmacokinetics of N-acetylcysteine in man. Eur J Clin Pharmacol. 31:217-22.
Duan M, Qiu J, Laurell G, Olofsson A, Counter SA, Borg E. (2004) Dose and time-dependent protection of the antioxidant N-L-acetylcysteine against impulse noise trauma. Hearing Research 192:1-9.
Knudsen TT, Thorsen S, Jensen SA, Dalhoff K, Schmidt LE, Becker U, Bendtsen F. (2005) Effect of intravenous N-acetylcysteine infusion on haemostatic parameters in healthy subjects. Gut 54:515-21
Kopke R, Bielefeld E, Liu J, Zheng J, Jackson R, Henderson D, Coleman JK. (2005) Prevention of impulse noise-induced hearing loss with antioxidants. Acta Otolaryngol 125:235-43
KS-OAS1 EudraCT 2006-006243-31 8 (8)
Kramer S, Dreisbach L, Lockwood J, Baldwin K, Kopke R, Scranton S, O'Leary M (2006). Efficacy of the antioxidant N-acetylcysteine (NAC) in protecting ears exposed to loud music. J Am Acad Audiol 17:265-78.
Meyer SE. (1999) The effect of stapes surgery on high frequency hearing in patients with otosclerosis. Am J Otol. 20:36-40
Ohinata Y, Miller JM, Schacht J. (2003) Protection from noise-induced lipid peroxidation and hair cell loss in the cochlea. Brain Res 966:265-73
Schmidt LE, Dalhoff K. (2001) Risk factors in the development of adverse reactions to N-acetylcysteine in patients with paracetamol poisoning. Br J Clin Pharmacol. 51:87-91
Yamashita D, Jiang HY, Le Prell CG, Schacht J, Miller JM. (2005) Post-exposure treatment attenuates noise-induced hearing loss. Neuroscience. 134:633-42.

Summary of substantial amendments to the study protocol

November, 2007	An agreement was signed with the Karolinska Trial Alliance, an independent organization supporting clinical trials in the Stockholm area. The Karolinska Trial Alliance will monitor the study throughout the duration of the trial.
June, 2008	Page 3 of the study protocol states that the infusion of N-Acetylcysteine starts one hour before surgery. Because of difficulties in guaranteeing that surgery starts at exactly the time planned, the study protocol was amended. The amendment states that the infusion should start as close to one hour before surgery as can practically be achieved.
February, 2009	Based on a per-protocol analysis of blinded data from the first 30 patients, the sample size was increased from 110 to 150 patients. Note that this analysis was performed without knowledge of treatments assignments and that no hypothesis testing was performed. The amendment also specifies that the interim analysis would take place after the recruitment of 75 patients, rather than 55 as initially planned. Because of slower than expected recruitment, two additional sites were included (Academic Hospital, Uppsala, and Karolinska University Hospital Huddinge). These changes were approved by the Regional Ethics Board and by the Swedish Medical Products Agency.
May, 2009	The protocol specifies that blinded data from the first 30 patients should be used for developing the statistical model. The treatment assignments of all patients remained concealed throughout this analysis and no hypothesis testing was performed. Based on this per-protocol analysis, statistical consultants recommended the use of linear mixed models for analyzing study data, rather than multiple linear regression.
